# Perception of Menstrual Normality and Abnormality in Spanish Female Nursing Students

**DOI:** 10.3390/ijerph17176432

**Published:** 2020-09-03

**Authors:** Ana Abreu-Sánchez, María Laura Parra-Fernández, María Dolores Onieva-Zafra, Elia Fernández-Martínez

**Affiliations:** 1 Department of Nursing, University of Huelva, 21071 Huelva, Spain; abreu@denf.uhu.es; 2 Department of Nursing, Physiotherapy and Occupational Therapy, University of Castilla-La-Mancha, Ciudad Real, 13071 Ciudad Real, Spain; marialaura.parra@uclm.es (M.L.P.-F.); mariadolores.onieva@uclm.es (M.D.O.-Z.)

**Keywords:** disorders menstrual, menstrual experiences, menstrual health

## Abstract

Menstrual problems are usually taboo; and often, some, such as dysmenorrhea, are presumed normal. This study seeks to compare the menstrual characteristics and symptoms of female university students reporting self-perceived normality concerning their cycles and menstruation with those who perceive their menstruation as being abnormal. A cross-sectional descriptive study was conducted among 270 nursing students using a self-report questionnaire that included sociodemographic and gynecological issues, together with Visual Analog Scale, the Andersch and Milsom Scale, and the Spanish version of the EuroQol-5 Dimension (EuroQol-5D) to measure self-perceived health status. A bivariate analysis was performed using the chi-square test, linear trend chi-square, and Student’s t-test, and a multivariate analysis of stepwise binary logistic regression was performed to predict the perception of cycle abnormality. In total, 77.4% of participants displayed normality; however, in self-reporting of menstrual characteristics, 67.4% identified alterations. Young women suffering from menstrual dizziness were 1.997 (CI95% = 1.010–3.950; *p* = 0.047) more likely to manifest abnormal menstruation, 4.518 (CI95% = 1.239–16.477; *p* = 0.022) more likely if they suffered from Grade 3 menstrual pain, and 2.851 (CI95% = 1.399–5.809; *p* = 0.004) more likely if they perceived that menstruation interfered with their daily lives. Many menstrual changes and symptoms are still considered normal, making it difficult to identify and address these issues. Therefore, it is necessary to develop health policies and strategies to improve menstrual health literacy for increased knowledge and earlier diagnosis.

## 1. Introduction

Normal menstruation is considered to occur every 24 to 38 days, on a regular basis, with bleeding that lasts 4.5 to 8 days, and 5 to 80 mL blood loss per cycle [1,2]. During the first menstrual cycle after menarche, alterations affecting the menstrual cycle are common, and are considered characteristics of the immaturity of the hypothalamic-pituitary-gonadal system. This is usually manifested by irregular cycles and anovulatory cycles [3]. In recent years, numerous studies have focused on the study of menstrual problems in young people, because, although they are usually not serious, they can have an impact on their daily activities and repercussions at the academic level [3,4,5,6,7]. The main gynecological problem affecting women of childbearing age is dysmenorrhea, for which two types exist: primary dysmenorrhea (which is not associated with any diagnosed pelvic pathology), and secondary dysmenorrhea (which is related to an identified cause) [8]. Primary dysmenorrhea is the most common form, which usually manifests at menarche, or a few months later, and is mainly related to an excess of prostaglandins, and can be accompanied by various other symptoms, such as nausea, vomiting, diarrhea, tiredness, or insomnia [8]. A recent international meta-analysis estimated the prevalence of dysmenorrhea in university students at 74.9% (CI95% 62.9–84.0) [4]. In Spain, the prevalence of dysmenorrhea has been estimated at 73.8–74.8% among university students [9,10]; corresponding to about 63.3% affected by primary dysmenorrhea and 10.5% by secondary dysmenorrhea [10]. In addition, other menstrual disorders, such as hypermenorrhea, menstrual irregularity, and other menstrual symptoms are less explored, both internationally and in Spain [11,12]. The aims of the published studies are very heterogeneous, although most focus on the prevalence, risk, protective factors, and impacts on their lives [4,12,13,14].

Previous studies have identified that young women with menstrual problems often do not consult health professionals for a variety of reasons, including normalization of disturbances, feelings of shame, lack of trust in the health professional, and lack of knowledge of menstrual normality [14,15,16]. This phenomenon of avoidance of consultation for menstrual problems is observed in young people worldwide [17], and also, specifically, in Spain [9,18], identifying a high percentage of women who self-medicate to address their menstrual problems [18]. Along these lines, the literature points out the need for early diagnosis and treatment [19], which requires prior literacy training for the population on menstruation (through educational interventions) [20].

In all territories, aspects related to menstruation have traditionally been immersed in multiple myths and taboos, some of which persist today [21]. In addition, socially, there is still a tendency to conceal menstruation, as reflected in television adverts for menstruation-related products [22]. Seear [23] even pointed out that publicizing menstrual issues can make women more vulnerable to stigmatization, and various authors in the same vein highlight the need to implement new educational strategies in this regard [24,25].

Studies on the perception of menstrual normality are scarce, and no study to date has examined the perception of menstrual normality by young Spanish women, which could serve as a starting point to guide future literacy studies on menstrual health to contribute towards female empowerment.

The present study aimed to compare the menstrual characteristics and symptoms of female university students who report self-perceived normality concerning their cycles and menstruation with those who perceive abnormality.

## 2. Materials and Methods 

### 2.1. Design

Cross-sectional descriptive study.

### 2.2. Participants and Sample

The inclusion criteria were women between the ages of 18 and 25 enrolled in the 2019–2020 nursing degree program at the University of Andalusia, Spain. The exclusion criteria were women with chronic or acute health problems manifesting as pain, women with diagnosed gynecological pathology, those who had undergone surgery for some gynecological and/or pelvic problem, and those who did not meet the inclusion criteria.

### 2.3. Study Variables and Data Collection

Data collection was carried out by means of an ad hoc paper questionnaire based on previous studies on menstrual problems. The questionnaire included socio-demographic issues, gynecological issues, and menstrual symptoms. 

Anthropometric data were collected through self-reports of participants’ weight and height, which allowed the calculation of the Body Mass Index (BMI). The main study variable was a closed-ended question concerning the self-perceived normality of a woman’s menstrual cycle: do you consider your menstrual cycles to be normal or, on the contrary, do you perceive any or all menstrual characteristic(s) or symptom(s) to be abnormal? (Normal/Abnormal). The Visual Analog Scale (VAS) was included to evaluate the self-perceived intensity of menstrual pain from 0 to 10 in women who experienced suffering from menstrual pain; this was categorized according to previous studies, into mild (0–3) and moderate–severe (4–10) [9,26,27]. To identify the perceived health status, the Spanish version of the EuroQol-5 Dimension (EuroQol-5D) was included, which includes a vertical graph with the VAS from 0 to 100, in which 0 is the worst imaginable health status and 100 is the best imaginable health status [5,28,29]. The Andersch and Milsom Scale was also used to determine the severity of dysmenorrhea according to the following grades: grade 0 (menstruation is not painful and daily activity is unaffected), grade 1 (menstruation is painful but seldom inhibits normal activity, analgesics are seldom required and mild pain), grade 2 (daily activity affected, analgesics required and give relief so that absence from work or school is unusual and moderate pain), and grade 3 (activity clearly inhibited, poor effect of analgesics, vegetative symptoms, and severe pain). Four closed questions were included on: pain intensity (not painful/mild pain/moderate pain/severe pain), working ability (unaffected/rarely affected/moderately affected/clearly affected/clearly inhibited), systemic symptoms (none/few/apparent), need for analgesia (not required/rarely required/required/poor effect) [18,30,31,32,33]. Normal menstrual characteristics were considered regular cycles of 24 to 38 days, with bleeding lasting 4.5 to 8 days, and with 5 to 80 mL blood flow [1,2]. As the amount of menstrual bleeding is difficult to quantify, students were asked to consider the number of pads used per day, light (≤5 pads/day), medium (5–7 pads/day), or heavy (≥7 pads/day). A light or medium amount of flow was considered normal, whereas heavy was considered abnormal [6,18].

### 2.4. Ethics

This study was approved by the Andalusia Research Ethics Committee (Code: 1013-N-19). Prior to the study, all participants signed the informed consent. Furthermore, the principles of the Declaration of Helsinki and the current data protection regulations were respected at all times.

### 2.5. Statistical Analysis

A descriptive analysis was performed in which quantitative variables were described by means and standard deviation, whereas qualitative variables were described using frequencies and percentages. A bivariate analysis was conducted using the chi-square test for comparison of categorical variables in women who reported that their cycles were normal, and those who considered their cycles as being abnormal. Moreover, the Student’s t-test was used for comparison in the same groups of continuous quantitative variables. To compare self-perceived normality in women with different degrees of menstrual pain, the Chi-square for linear trends was used.

Finally, an age-adjusted, stepwise binary logistic regression was performed to predict the perception of cycle abnormality from the significant variables identified in the bivariate analysis. The significance level was set at 0.05.

## 3. Results

### 3.1. Sociodemographic, Anthropometric, and Gynecological Results

In total, 270 young university women participated in the study, with an average age of 20.63 ± 1.78 years, weight of 60.07 ± 9.29, height of 164.10 ± 6.23, and BMI of 22.30 ± 3.15 kg/m^2^. Of these, 75.4% resided in an urban environment.

The mean self-perceived health status was 80.20 ± 11.69. Regarding gynecological characteristics, the mean age of menarche was 12.14 ± 1.51 years. Seventy-three per cent of participants reported a regular cycle, 79.8% a light–moderate amount of bleeding, 87.4% reported that their menstrual cycles were between 24 and 38 days, 63.7% referred that each cycle had one day of menstrual bleeding, and 21% reported that their menstruation was not painful. Finally, 28.5% reported using oral contraceptives (OCPs).

### 3.2. Perception of Normal Menstruation and Sociodemographic and Anthropometric Characteristics

No differences were found when comparing age, weight, height, BMI, and perceived health status between women with self-perceived normal menstruation and those with abnormal menstruation. The proportion of women who perceived abnormality was slightly higher in urban women (20.2%) than in rural women (19%) (*p* > 0.05).

### 3.3. Self-Perceived Normal Menstruation and Menstrual Characteristics

Up to 77.4% perceived their menstruation as normal and 19.6% stated that they did not have a normal menstruation in spite of not having been diagnosed with any gynecological problem. 

Among the women who reported having normal menstruation, only 42.6% had normal menstrual characteristics in terms of regularity, frequency, duration, and blood flow, based on the analysis of responses to specific questions regarding menstrual characteristics. Therefore, 67.4% claimed to have normal menstruation; however, in their specific self-reports, concerning the characteristics of their menstrual cycles, some degrees of abnormalities were identified.

The mean age of menarche was similar in women who perceived normality (12.14 ± 1.48) versus those who did not (12.06 ± 1.52) (*p* > 0.05). 

Table 1 shows the comparison of menstrual characteristics in women with self-perception of normality and menstrual abnormality. Among women with an abnormal cycle length, there was also a higher percentage (33.3%) of those who considered their menses to be abnormal than among those with normal cycle length (18.3%) (*p* = 0.045). Comparing the mean cycle length between women with self-perceived normal and abnormal cycle lengths, a higher mean cycle length was identified in those with perceived abnormality (30.92 ± 7.54 vs. 28.44 ± 3.62; *p* = 0.023).

Of the total participants who perceived normality, 56.6% had an irregular cycle as opposed to 43.4% who had a regular cycle (*p* < 0.01).

### 3.4. Normal Menstrual Perception and Menstrual Symptoms

Of the total number of women who reported abnormal menstruation, 90.6% suffered from menstrual pain compared to 9.4% of those who did not report such pain (*p* = 0.021). However, of those who considered their menstruation to be normal, 76.1% also suffered from menstrual pain.

Women with menstrual pain were more frequently considered to have abnormal menstruation among those with Grade 3 pain (66.7%) than in those with Grade 2 (25.4%), Grade 1 (9.7%), and Grade 0 (9.1%) pain (*p* < 0.01). In relation to the intensity of menstrual pain when evaluated on the VAS Scale and categorized according to previous studies, 96.2% of women with moderate–severe pain intensity considered their menstrual cycle to be abnormal compared to 5.6% of those with more mild pain (*p* = 0.018). 

Up to 98.1% of the women who considered their menstruation to be abnormal complained of tiredness during menstruation, compared to 1.9% of those who did not feel tired (*p* = 0.012). Moreover, 58.5% of those who showed abnormality perceived a decrease in concentration on the days of menstruation, which was higher than the percentage of those who did not perceive any alteration in their concentration (41.5%) (*p* = 0.009). 

Among the women who suffered from dizziness during menstruation, in Table 2 it is shown that 31.1% considered their menstruation abnormal; however, among those who did not suffer from this symptom, only 16% considered it abnormal (*p* = 0.006).

Of the total number of women who had to miss classes or clinical practice during their menstruation due to menstrual symptoms, only 25.2% considered their period to be abnormal. Moreover, of the total number of women who considered their period to be abnormal, 71.7% had missed some amount of class time or clinical practice training in the last year due to their period.

### 3.5. Logistic Regression to Predict the Perception of Abnormal Menstruation

Table 3 summarizes regression data to predict perceived menstrual abnormality from menstrual symptoms and characteristics. Women who suffered from dizziness during menstruation were identified as being 1.997 (CI95% =1.010–3.950; *p* = 0.047) times more likely to manifest abnormal menstruation, 4.518 (CI95% = 1.239–16.477; *p* = 0.022) times more likely if they suffered Grade 3 menstrual pain and 2.851 (CI95% = 1.399–5.809; *p* = 0.004) times more likely if they perceived that menstruation interfered with their daily lives.

## 4. Discussion

Up to 77.4% of participants stated that their menstruation and menstrual cycle was normal; however, upon answering more specific questions, 67.4% stated abnormal characteristics. Having an irregular and abnormal cycle length were the cycle characteristics most commonly associated with self-perceived abnormality, the remaining alterations were perceived as normal in most young women. 

In terms of symptoms, nausea, dizziness, tiredness, lack of concentration, and menstrual pain were most prevalent in women who reported an abnormal cycle. However, these findings also highlight that 76.1% of women who considered their cycle to be normal also suffered from menstrual pain. The perception of an abnormal cycle was also more frequent in women who stated that the pain appeared or intensified when walking, with a full bladder, when menstrual issues caused them to miss clinical practices, and when it interfered with their daily lives and social lives.

The regression showed that young women who suffered from menstrual dizziness were 1.997 (IC95% = 1.010–3.950; *p* = 0.047) times more likely to report abnormal menstruation, 4.518 (IC95% = 1.239–16.477; *p* = 0.022) times more likely if they had Grade 3 menstrual pain and 2.851 (IC95% = 1.399–5.809; *p* = 0.004) times more likely if they perceived that menstruation interfered with their daily life.

The fact that more than half of the women (67.4%) who reported normal menstruation had some kind of disorder shows that health science students are not aware of the normal characteristics of the menstrual cycle or are not able to apply the definition to their own health assessment. This is in line with previous findings, considering that the participants were nursing students and, therefore, had greater knowledge of gynecological health issues than the rest of the population [34]. Thus, it is assumed that higher percentages may be found in university students from other disciplines or in women without university studies. Therefore, there is a need to explore women’s knowledge of menstrual normality in future studies, independently of their self-evaluation. Moreover, it seems necessary to implement educational strategies along these lines, because young women being able to identify their menstrual disorders is the first step for them to seek consultation with health professionals. Irregularity and abnormality in cycle length were the two menstrual characteristics associated with perception of abnormality, coinciding with those identified in previous studies in other countries and among young women in Spain [4,12,13,14]. It is likely that these characteristics may be of more concern to college girls as both characteristics may be associated with delayed menstruation, and there is also the possibility of an unwanted pregnancy, which is often a concern for female university students [35].

Menstrual pain was reported by 90.6% of those who expressed self-perceived abnormality of their menstrual cycle. Regarding the intensity of menstrual pain, 96.2% of women with moderate–severe intensity of menstrual pain manifested self-perceived abnormality. In relation to the degree of pain according to the Andersch and Milsom Scale, a higher percentage of women with perceived abnormality was identified with Grade 3 pain (66.7%). A Grade 3 on this scale indicates that activity is clearly inhibited, together with a poor effect of analgesics, vegetative symptoms, and severe pain. It seems logical that women with this degree of pain have a greater perception of abnormality. Along the same lines, previous studies have already identified that women with a higher degree or severity of menstrual intensity have a poorer quality of life and a higher rate of absenteeism during menstruation [5,12,31]. However, in the current study, no association was found between the consumption of OCPs and the perception of normality, although it was expected that such an association would be found according to the previous literature that related the consumption of OCPs to a lower degree of dysmenorrhea (the Andersch and Milsom Scale) [33], and less menstrual absenteeism [12]. Our results could be attributed to the fact that it is likely that some of the participants consumed these OCPs to manage their self-perceived menstrual abnormality, and although improvement during consumption is common, it is likely that they manifest that their menstrual cycles as such are not normal. It should be noted that previous studies indicate that self-medication without consulting health professionals in young people with menstrual disorders is very common [9,18] and, on the other hand, self-medication in general is also a frequent practice among nursing students [36,37]. Other symptoms more related to menstrual abnormality were nausea, dizziness, fatigue, and lack of concentration. Although it should be noted that some authors consider these symptoms related to menstrual pain itself, in our study, as in a previous one [12], we identified that they are also manifested in women who do not suffer from menstrual pain; therefore, it seems interesting to continue studying them individually, in women with and without dysmenorrhea. Nausea, dizziness, and menstrual pain had been previously linked to menstrual absenteeism in college women [12], which seems consistent with the results of this study, which identified a higher proportion of women with abnormalities who suffered from these symptoms and were absent from classes and/or clinical practices during menstruation.

The results of the regression have identified that women who suffer from dizziness, a Grade 3 in pain, and perceive that these symptoms interfere with their activities of daily living are more likely to perceive their cycles as being abnormal. This could be interpreted to mean that female perception of menstrual abnormalities are primarily based on whether or not they have very severe and limiting pain or symptoms that impact their daily lives, such as dizziness. All other abnormal characteristics and symptoms seem to go unnoticed by young women, because those who suffer from these characteristics do not usually manifest abnormality. When comparing these findings with the only study found on menstrual absenteeism among Spanish university women, there were coincidences in the detection of menstrual pain and dizziness as being more likely in women who are absent from classes and/or practical training during menstruation; however, in the former study, other symptoms were also identified as being more likely in women who missed class during their periods (nausea and vomiting, sleep disturbances, and feeling depressed) [12]. However, in the present study, only nausea was identified as a statistically more frequent symptom in those who considered their menstrual cycle to be abnormal. This variable did not enter the regression equation, suggesting that it should be further explored in future studies. Moreover, another study identified menstrual migraine as a factor related to menstrual absenteeism [38]. However, women with this symptom were not identified as having significantly higher perceptions of menstrual abnormalities in the current study, which may be due to the fact that young women may consider this menstrual symptom to be normal, as is the case with normalization of menstrual pain at Grade 0 and 1 [15].

The multiple myths and taboos related to menstruation that are still present in our society today are likely to be negatively influencing women’s menstrual health [39] and can be explained in the context of a gender gap in women’s health as self-silencing [40]. Although several studies have focused on the fact that women do not consult professionals for menstrual problems, our study assumes that perceiving cycle abnormality is the first step before consulting a health professional. The results of this study show that a high percentage of women with menstrual disorders are not aware of these and consider their cycles to be normal. Therefore, this highlights a significant knowledge gap in this regard that must be addressed, as reported in the qualitative study carried out by Wood et al. on the normality or abnormality of menstrual cycles in university women in Pennsylvania [41]. In addition, previous studies indicate that even women who perceive that their cycles are not normal usually fail to consult professionals as they believe that they will not assign any importance to these symptoms, or because of feelings of shame, or fear of being stigmatized by their close environments [15]. However, health professionals can assist these women in a number of ways, including reporting the available scientific evidence on lifestyle factors that positively and negatively influence menstrual characteristics and symptoms [11,42,43].

Regarding symptom relief, the most explored menstrual symptom in the literature is relief from menstrual pain [17,18,44]. Furthermore, it has been identified that most women self-medicate with sub-therapeutic doses, which results in an unsuccessful approach [45]. Health professionals have an important role to play in this menstrual problem, as they can individually evaluate and detect secondary dysmenorrhea, advise on risk factors and protective factors, on the correct pharmacological approach, as well as report on non-pharmacological methods with proven efficacy that have fewer side effects, such as those based on physical exercise [28,46]. However, young women usually receive information about menstruation within their own families, from their mothers or sisters, yet, they are not sufficiently prepared to meet their needs [47,48]. Along these lines, Wong et al. pointed out that both the young woman’s own menstrual education and the parents’ level of education and training in this regard influence young women’s menstrual behavior [49]. Currently, women also seek information on the internet due to its ease of accessibility, although the information on menstruation that these websites provide is often inaccurate and confusing [50]. For these reasons, in recent years, several authors have pointed out the need for greater menstrual literacy and have analyzed current educational interventions in this regard, stressing that these websites are having a poor effect in terms of dismantling taboos and menstrual myths [21,24,25]. It is therefore considered necessary to continue working on more effective interventions. These should be based on confidentiality, therapeutic communication, and culturally sensitive care, favoring women’s empowerment [39,51]. 

This study is the first to analyze the perception of menstrual normality and abnormality in Spanish women. It also provides the added value of comparing the menstrual characteristics and symptoms of both groups of women: those who perceive menstrual normality and those who do not, identifying the most influential aspects in the perception of abnormality. Furthermore, these findings highlight gaps in knowledge and/or self-evaluation among young people in relation to their menstrual normality or abnormality. Several studies indicate that the fact that women do not consult professionals regarding menstrual problems and decide to take care of themselves or self-medicate is a problem, as, in many cases this is inefficient management, or the problem is aggravated by the delay in consultation [15,18]. This study identifies a clear dissonance between the perceived abnormality and certain abnormal characteristics of the cycle when specifically analyzed, even though the group studied are students of health sciences. Thus, this population is expected to have more knowledge of the subject and greater ability to access scientific information related to health compared to other young people with lower levels of education. This underlines the need to improve the menstrual health literacy of all young women.

### Limitations

These results must be interpreted while considering certain limitations due to the cross-sectional nature of this study, carried out in students from a single Spanish faculty. Additionally, the fact that all participants were a convenience sample of nursing students implies a selection bias, which seems to suggest that students from other non-health related disciplines may have a lesser agreement between perceived normality and normal menstrual characteristics. Another limitation is that only women under the age of 25 were included, which is considered in much of the gynecological literature as young women [4,52,53]. While this favored the homogeneity of the sample, facilitating comparison with the results of other studies, it also limits the possibility of generalizing the results towards women in other age ranges. Additionally, women with gynecological problems were excluded, although women with endocrine problems were not excluded, except those with polycystic ovary syndrome. This should be taken into account when interpreting the results, since other endocrine alterations, such as thyroid disorders, can influence menstrual characteristics [54,55]. Thus, in future studies it would be interesting to collect and analyze this variable. However, this paper is a first step in identifying information gaps and encourages further research into developing menstrual health education strategies from early stages. It would also be interesting for future studies to deepen our understanding of the influence of OCP consumption by performing a sensitivity analysis.

## 5. Conclusions

Most students consider their menstrual cycles and menses to be normal. However, a high percentage of women who consider their menstrual cycles to be normal have abnormal characteristics, menstrual symptoms, or both. Considering that the participants were nursing students, this reveals a deficit in knowledge in this subject or ability to correctly self-evaluate oneself that may be higher, still, in students from other non-health related disciplines. Self-perceived abnormality is considered key for women in order to seek healthcare. Therefore, it is necessary to direct health policies and strategies to improve knowledge of menstrual health, raise awareness of menstrual problems, and promote a comprehensive approach among health professionals towards menstrual health among young women.

## Figures and Tables

**Table 1:** Comparison of menstrual characteristics in women with self-perception of normality and menstrual abnormality.

Menstrual Characteristics		Self-Perception of Your Menstrual Cycle		Total	*p*-Value ^a^
		Normal	Abnormal		
Regular menstrual cycle	No	42(58.3%)	30(41.7%)	72(27.5%)	0.000 *
	Yes	167(87.9%)	23(12.1%)	190(72.5%)	
Duration of cycle	Normal (24–38 days)	187(81.7%)	42(18.3%)	229(87.4%)	0.045 *
	Abnormal	22(66.7%)	11(33.3%)	33(12.6%)	
Duration of menses	Normal (4.5–8 days)	133(79.6%)	34(20.4%)	167(63.7%)	0.945
	Abnormal	76(80%)	19(20%)	95(36.3%)	
Amount of flow	Light	29(80.6%)	7(19.4%)	36(13.7%)	0.125
	Average	143(82.7%)	30(17.3%)	173(66%)	
	Heavy	37(69.8%)	16(30.2%)	53(20.2%)	
OCPs	No	150(78.5%)	41(21.5%)	191(72.9%)	0.414
	Yes	59(83.1%)	12(16.9%)	71(27.1%)	

^a^ Chi-square Test; * *p* < 0.05; OCPs: oral contraceptives.

**Table 2:** Comparison of menstrual symptoms in women with normal and abnormal menstrual periods.

Menstrual Symptoms		Self-Perception of Your Menstrual Cycle		Total *n* (%)	*p*-Value ^a^
		Normal *n* (%)	Abnormal *n* (%)		
Menstrual pain	No	50(90.9%)	5(9.1%)	55(21%)	0.021 *
	Yes	159(76.8%)	48(23.2%)	207(79%)	
Change in appetite	No	37(86%)	6(14%)	43(16.5%)	0.257
	Yes	171(78.4%)	47(21.6%)	218(83.5%)	
Nausea	No	159(84.6%)	29(15.4%)	188(71.8%)	0.002 *
	Yes	50(67.6%)	24(32.4%)	74(28.2%)	
Vomiting	No	193(80.8%)	46(19.2%)	239(91.9%)	0.124
	Yes	14(66.7%)	7(33.3%)	21(8.1%)	
Dizziness	No	158(84%)	30(16%)	188(71.8%)	0.006 *
	Yes	51(68.9%)	23(43.4%)	74(28.2%)	
Fatigue	No	30(96.8%)	1(3.2%)	31(11.8%)	0.012 *
	Yes	179(77.5%)	52(22.5%)	231(88.2%)	
Headache	No	97(84.3%)	18(15.7%)	115(43.9%)	0.103
	Yes	112(76.2%)	35(23.8%)	147(56.1%)	
Diarrhea	No	85(82.5%)	18(17.5%)	103(39.3%)	0.372
	Yes	124(78%)	35(22%)	159(60.7%)	
Constipation	No	181(80.1%)	45(19.9%)	226(86.3%)	0.749
	Yes	28(77.8%)	8(22.2%)	36(13.7%)	
Insomnia	No	166(80.2%)	41(19.8%)	207(79%)	0.741
	Yes	43(78.2%)	12(21.8%)	55(21%)	
Irritability	No	134(79.3%)	35(20.7%)	169(65.3%)	0.572
	Yes	74(82.2%)	16(17.8%)	90(34.7%)	
Depressive symptoms	No	67(82.7%)	14(17.3%)	81(31%)	0.415
	Yes	141(78.3%)	39(21.7%)	180(69%)	
Decreased concentration	No	128(85.3%)	22(14.7%)	150(57.3%)	0.009 *
	Yes	81(72.3%)	31(27.7%)	112(42.7%)	
Pain appears or is worse when sitting	No	45(86.5%)	7(13.5%)	52(21.3%)	0.137
	Yes	148(77.1%)	44(22.9%)	192(78.7%)	
Pain appears or gets worse when you put on weight	No	116(82.9%)	24(17.1%)	140(57.4%)	0.094
	Yes	77(74%)	27(26%)	104(42.6%)	
Pain appears or is worse when walking	No	83(88.3%)	11(11.7%)	94(38.7%)	0.005 *
	Yes	109(73.2%)	40(26.8%)	149(61.3%)	
Need to stop and sit down	No	87(87.9%)	12(12.1%)	99(37.8%)	0.011 *
	Yes	122(74.8%)	41(25.2%)	163(62.2%)	
Painful bowel movements	No	149(81.4%)	34(18.6%)	183(76.3%)	0.206
	Yes	42(73.7%)	15(26.3%)	57(23.8%)	
Pain with a full bladder	No	120(87%)	18(13%)	138(57.7%)	0.001 *
	Yes	70(69.3%)	31(30.7%)	101(42.3%)	
Painful urination	No	153(80.5%)	37(19.5%)	190(79.2%)	0.480
	Yes	38(76%)	12(24%)	50(20.8%)	
Pain with sex	No	153(80.5%)	37(19.5%)	190(77.9%)	0.303
	Yes	40(74.1%)	14(25.9%)	54(22.1%)	
Missing classes or practices	No	96(86.5%)	15(13.5%)	111(42.4%)	0.020 *
	Yes	113(74.8%)	38(25.2%)	151(57.6%)	
Interferes with your daily life	No	113(89.7%)	13(10.3%)	126(48.3%)	0.000 *
	Yes	95(70.4%)	40(29.6%)	135(51.7%)	
Interferes with your social activities	No	122(85.9%)	20(14.1%)	142(54.2%)	0.007 *
	Yes	87(72.5%)	33(27.5%)	120(45.8%)	

^a^ Chi-square Test; * *p* < 0.05.

**Table 3:** Binary logistic regression to predict the perception of abnormal menstrual periods from menstrual characteristics and symptoms reported by women.

Menstrual Characteristics and Symptoms	OR ^a^	CI95%		*p*-Value
Grade 3	4.518	1.239	16.477	0.022 *
Dizziness	1.997	1.010	3.950	0.047 *
Interferes with your daily life	2.851	1.399	5.809	0.004 *

OR ^a^: Odds ratio age-adjusted; * *p* < 0.05.
